# Supplementary material for: Content-rich biological network constructed by mining PubMed abstracts
Source: BMC Bioinformatics. 2004 Oct 8;5:147. doi: 10.1186/1471-2105-5-147 (PMC528731; doi:10.1186/1471-2105-5-147)
Supplement: Additional File 2 — The original results of the above study (non-essential files are deleted to keep the file size under the limit set by BMC bioinformatics). [file 1471-2105-5-147-S2.bz2 › chilibotAdditionalFile2/dip10/412ID10712517E10078/html/BMPR1B_BMPR1A.html]

 


 **BMPR1B** and **BMPR1A** 
  
Found 25 abstracts in PubMed, retrieved 10.  
 

 What does Google say? 
 PDF only 
| .edu only 

---

**Interactive relationship** (e.g. stimulation, inhibition, etc)

**Neutral relationship**- BMP 4 and GDF 5 are known to bind to activin receptor like kinase 3  [ **BMPR1A** ]  ALK 3  [ **BMPR1A** ]  and or ALK 6  [ **BMPR1B** ]  also termed BMP type IA and type IB receptors, respectively.  Ref: 11282024 J Cell Sci, 2001
- Constitutively active BMP type I receptors, ALK 3  [ **BMPR1A** ]  and ALK 6  [ **BMPR1B** ] , as well as ALK 2, were phosphorylated Smad8 and induced Smad8 interaction with Smad4.  Ref: 10814522 Biochem Biophys Res Commun, 2000
- The ALK 3  [ **BMPR1A** ]  mRNA began to appear in this stage, as did ALK 6  [ **BMPR1B** ]  and BMP RII.  Ref: 11147870 J Neurosurg, 2001

**Non-interactive relationship** (e.g. studied together, co-existance, homology, etc.)

- Compared with the effects induced by only one of the type I receptors, the combination of constitutively active forms of ALK 2 and ALK 3  [ **BMPR1A** ]  or ALK 6  [ **BMPR1B** ]  more strongly induced alkaline phosphatase activity in C2C12 cells.  Ref: 11282024 J Cell Sci, 2001
- We assessed the distribution and relative immunohistochemical staining intensity of the bone morphogenetic protein 7, osteogenic protein 1 OP 1, in its pro and mature forms, and four of its receptors, type I ALK 2, ALK 3  [ **BMPR1A** ] , and ALK 6  [ **BMPR1B** ]  and type II in normal adolescent New Zealand White rabbit articular cartilage.  Ref: 12364567 J Histochem Cytochem, 2002
- A mechanistic explanation for increased pSmad1 5 staining during inflammation was provided by the upregulated expression of all the BMP type I receptors, i.e., activin receptor like kinase ALK 2, ALK3  [ **BMPR1A** ] , and ALK6  [ **BMPR1B** ] , in the inflamed bronchial epithelial cells.  Ref: 12151307 Am J Respir Cell Mol Biol, 2002
- Activin receptor like kinase ALK 1, ALK 2, ALK 3  [ **BMPR1A** ] , and ALK 6  [ **BMPR1B** ]  were able to reproduce the hypertrophic maturation of chondrocytes induced by BMP 2.  Ref: 12082094 J Biol Chem, 2002
- Thus, ALK 2 and ALK 3  [ **BMPR1A** ]  or ALK 6  [ **BMPR1B** ]  might synergistically induce osteoblast differentiation of C2C12 cells, possibly through efficient activation of downstream signaling pathways.  Ref: 11282024 J Cell Sci, 2001
- Alk3  [ **BMPR1A** ]  tended to decrease, but Alk6  [ **BMPR1B** ]  remained unchanged.  Ref: 11675415 J Am Soc Nephrol, 2001
- The induction of Tlx2 luc was dependent upon the kinase activity of MISRII and was blocked by a dominant negative truncated ALK2 tALK2 receptor but not by truncated forms of the other BMP type I receptors ALK1, ALK3  [ **BMPR1A** ] , or ALK6  [ **BMPR1B** ] .  Ref: 11376113 Mol Endocrinol, 2001
- The sections were rehydrated, submitted to microwave antigen retrieval and treated with polyclonal antibodies against  **BMPR1A** ,  **BMPR1B**  and BMPR2.  Ref: 11882013 Reproduction, 2002
- Mouse smad8 phosphorylation downstream of BMP receptors ALK 2, ALK 3  [ **BMPR1A** ] , and ALK 6  [ **BMPR1B** ]  induces its association with Smad4 and transcriptional activity.  Ref: 10814522 Biochem Biophys Res Commun, 2000
- Messenger RNAs for BMP, 7, the BMP type I receptors Act R 1 alk 2, activin like kinase receptor, BMPR IA alk 3  [ **BMPR1A** ] , IB alk 6  [ **BMPR1B** ] , and the type II receptor BMPR II were detected.  Ref: 11829442 Biomaterials, 2002
